# Effect of Vacancy Behavior on Precipitate Formation in a Reduced-Activation V−Cr−Mn Medium-Entropy Alloy

**DOI:** 10.3390/ma16010153

**Published:** 2022-12-23

**Authors:** Tianjiao Wang, Te Zhu, Dandan Wang, Peng Zhang, Yamin Song, Fengjiao Ye, Qianqian Wang, Shuoxue Jin, Runsheng Yu, Fuyan Liu, Peng Kuang, Baoyi Wang, Liben Li, Xingzhong Cao

**Affiliations:** 1 School of Physics and Engineering, Henan University of Science and Technology, Luoyang 471023, China; 2 Institute of High Energy Physics, CAS, Beijing 100049, China; 3 Department of Mechanical Engineering, The University of Hong Kong, Pokfulam Road, Hong Kong, China

**Keywords:** vacancy, precipitate, positron annihilation lifetime, coincidence Doppler broadening

## Abstract

In this work, we studied the evolution of vacancy-like defects and the formation of brittle precipitates in a reduced-activation V−Cr−Mn medium-entropy alloy. The evolution of local electronic circumstances around Cr and Mn enrichments, the vacancy defects, and the CrMn_3_ precipitates were characterized by using scanning electron microscopy with energy-dispersive spectroscopy, X-ray diffraction, and positron annihilation spectroscopy. The microstructure measurements showed that the Mn and Cr enrichments in the as-cast sample significantly evolved with temperature, i.e., from 400 °C, the Cr/Mn-segregated regions gradually dissolved into the matrix and then disappeared, and from 900 °C to 1000 °C, they existed as CrMn_3_ precipitates. The crystallite size of the phase corresponding to CrMn_3_ precipitates was about 29.4 nm at 900 °C and 43.7 nm at 1000 °C. The positron annihilation lifetime results demonstrated that the vacancies mediated the migration of Cr and Mn, and Cr and Mn segregation finally led to the formation of CrMn_3_ precipitates. The coincidence Doppler broadening results showed that the characteristic peak moved to the low-momentum direction, due to an increase in the size of the vacancy defects at the interface and the formation of CrMn_3_ precipitates.

## 1. Introduction

Structural materials for Gen-IV fission and fusion nuclear power plants operate in harsh working conditions such as intense neutron radiation and high temperatures, experience time-varying stress and corrosive environments, and require environmentally friendly strategies (e.g., reducing the export of high-level radioactive waste). This has spurred the worldwide research of advanced nuclear reactor systems, especially nuclear energy structural materials. Reduced neutron activation is one of the key issues in the development of nuclear structural materials, and reduced-activation materials play key roles in the development of advanced nuclear power systems to achieve environmental safety and social acceptability [1,2,3,4]. In the last three decades, many reduced-activation materials have been developed, such as reduced-activation ferritic/martensitic steels (RAFMs) [5,6] and vanadium alloys [7,8], which meet low-activation requirements and offer good room-temperature performance and high irradiation resistance. However, they still require significant improvement before they can be used in advanced nuclear energy systems.

Medium-entropy alloys (MEAs), formed by mixing low-activation elements and high-melting elements, can absorb the original characteristics of elements, resulting in high strength, excellent radiation resistance, and high-temperature softening resistance, and can withstand nuclear reactor environments [2,9,10,11,12]. As a candidate structural material for nuclear power systems, vanadium alloys can achieve the best low-activation characteristics and irradiation performance [8,13,14]. By absorbing these excellent characteristics in a vanadium base alloy, Carruthers et al. designed reduced-activation TiVCrFe-based high-entropy alloys (HEAs) with a body-centered cubic (BCC) structure and reported their mechanical strength and toughness [9]. The researchers found that these alloys had high strength and hardness, but their ductility was limited due to the presence of brittle precipitates. For structural applications in a nuclear reactor, ductility is an important evaluation index of alloy materials. The ductility of BCC H/MEAs will be largely affected by brittle precipitates such as the precipitates from the Laves phase. Therefore, to improve the ductility of reduced-activation BCC HEAs and realize their application in nuclear power systems, it is necessary to gain an in-depth understanding of the formation and evolution mechanisms of these precipitates [4,15,16].

V and Cr, with high melting points, offer good creep performance at high temperatures, and Cr can improve the oxidation resistance of materials [7,14,15]. In addition, Mn can increase the configuration entropy of the solid solution and improve an alloy’s resistance to high-temperature stability, possibly enabling the introduction of a higher element concentration [8,17,18]. The development of new HEAs based on V−Cr−Mn offers good prospects for realizing reduced-activation HEAs for nuclear applications [7].

In this work, we fabricated a reduced-activation V−Cr−Mn MEA with a BCC structure in the matrix and studied the effect of vacancy-defect evolution on the formation and evolution of the precipitates. Studying the formation of precipitates can help to solve the problem of poor ductility and the brittleness of BCC alloys. Therefore, the interactions between the vacancy-type defects and solute atoms during the isochronous annealing of the V−Cr−Mn cast alloys, as well as their influence on the formation of CrMn_3_-precipitated phases, were studied via positron annihilation spectroscopy.

## 2. Materials and Methods

### 2.1. Sample Preparation

The equiatomic material V−Cr−Mn (the V, Cr, and Mn elements have the same atomic percentage content) used in this work was obtained through the arc-melting of high-purity (99.99%) V, Cr, and Mn metals. Samples 10 × 10 × 0.5 mm^3^ in size were cut from ingots and polished with SiC sandpaper up to 5000 grit, and then diamond spray was used to mechanically polish the samples’ surfaces to obtain a mirror-like state. Subsequently, electrochemical polishing was used in a 3:1 solution of acetic acid and perchloric acid under a constant current of 0.6 A, as the finishing step to remove the damaged layer caused by mechanical polishing and surface enrichment due to the different rates of electrochemical oxidation of ingredients. Isochronal annealing was carried out for 0.5 h in the $5.0\times 10^{-5}\;Pa$ vacuum for the prepared samples, where the annealing temperatures ranged from 100 to 1000 °C, with incremental steps of 100 °C.

### 2.2. Experimental Methods

After each annealing treatment, positron annihilation lifetime (PAL) spectra, coincidence Doppler broadening (CDB) spectra, X-ray diffraction (XRD), and scanning electron microscopy with energy-dispersive spectroscopy (SEM-EDS) analyses were performed on the samples in this work.

The PAL spectra enabled the identification of vacancy defects and revealed the relative information on the number density of defects (in case no saturation of positron trapping was observed in the defects), while the CDB spectra could provide information on the local chemical environment regarding the defects. PAL analysis in this work was performed using a fast–slow coincidence system with a temporal resolution of about 200 ps (FWHM). Two pieces of identical samples were sandwiched together with a 10 µCi ^22^Na positron source, and each spectrum contained 10^6^ counts. The CDB spectra were measured using two Ge detectors, which were used to measure the momentum distribution of the core electrons from the V, Cr, and Mn atoms in this work, with the results shown by the CDB ratio spectra. XRD analysis, typically used to determine the crystal structures of alloys, was carried out by using Cu-Kα radiation on a D8 Advance X-ray diffractometer, and the data were collected from 30° to 100° (2θ) with a step size of 3°/min. SEM was carried out on a SU8020 instrument with EDS to obtain back-scattered electron images with a magnification of 1 K for the as-cast samples and the samples annealed at 400 °C and 700 °C.

## 3. Results and Discussion

**Figure 1:**
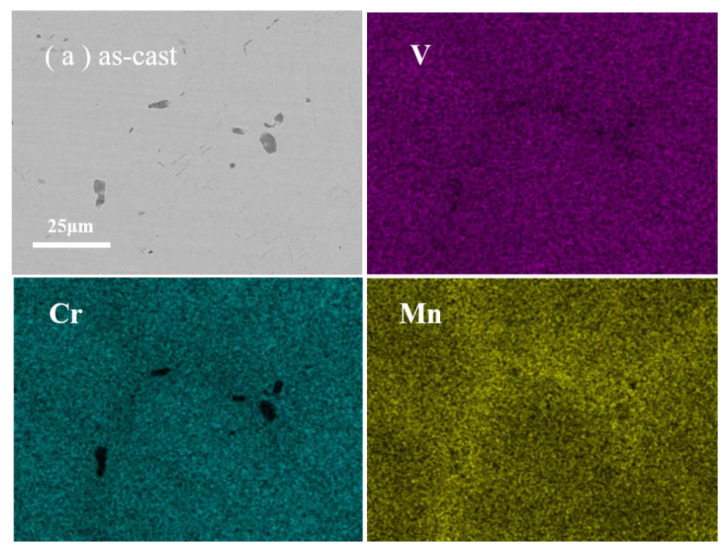

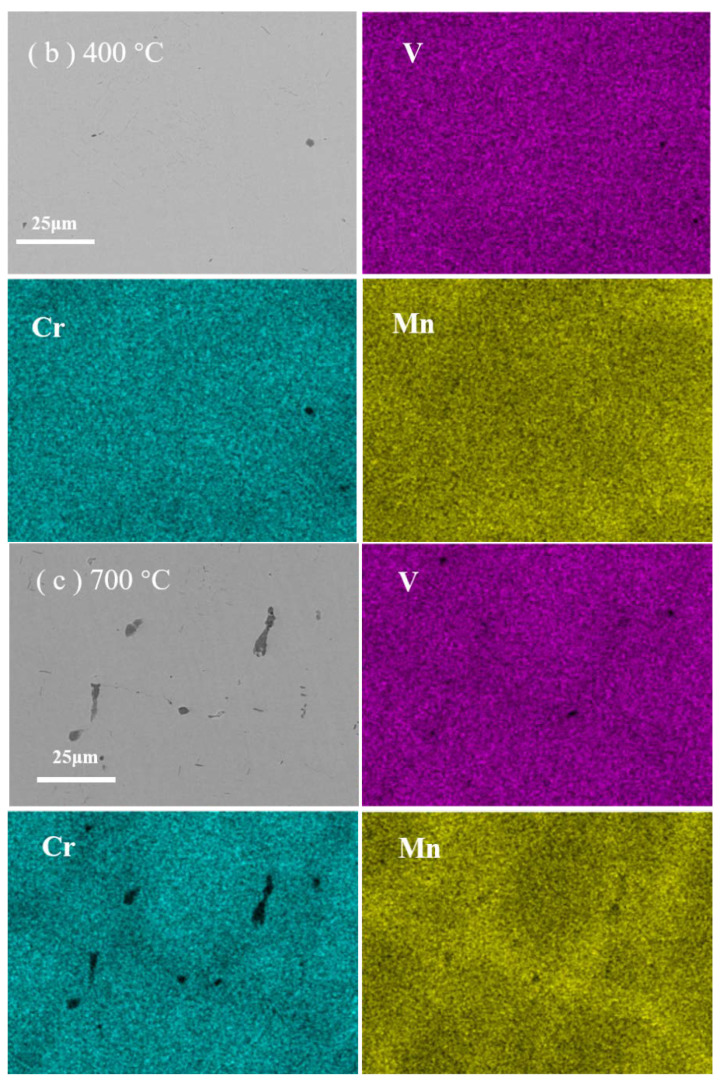
The SEM images and EDS mappings of (**a**) the as-cast V−Cr−Mn alloy, (**b**) V−Cr−Mn alloy after annealing at 400 °C, and (**c**) V−Cr−Mn alloy after annealing at 700 °C.

Figure 1 shows the SEM images and EDS mappings of the V−Cr−Mn alloy in the as-cast samples, after annealing at 400 °C and 700 °C. In the as-cast alloy, the contents of the three elements (V, Cr, and Mn in order) were 37.72 at%, 37.29 at%, and 24.99 at%, respectively. In the sample annealed at 400 °C, the contents were 38.72 at%, 38.91 at%, and 22.37 at%. Additionally, in the sample annealed at 700 °C, the contents were 36.49 at%, 35.99 at%, and 27.5 at%, respectively. We observed a slight level of Mn segregation in the grain boundaries and a Cr-rich region in the matrix of the as-cast alloy. At 400 °C, the Mn-segregated region disappeared, and the Cr-rich region was uniformly distributed in the alloy, indicating that the Mn-segregated region dissolved, and the Mn-rich region migrated into the matrix. This indicated the migration of Cr and Mn solutes, and this migration would induce vacancies [19]. Notably, Mn segregation occurred in the grain boundaries, and Cr segregation occurred in the matrix again at 700 °C. Therefore, from 400 °C, Cr and Mn solutes continued to migrate and form secondary aggregates and then underwent segregation. This process was also accompanied by the continuous generation of vacancies.

**Figure 2:**
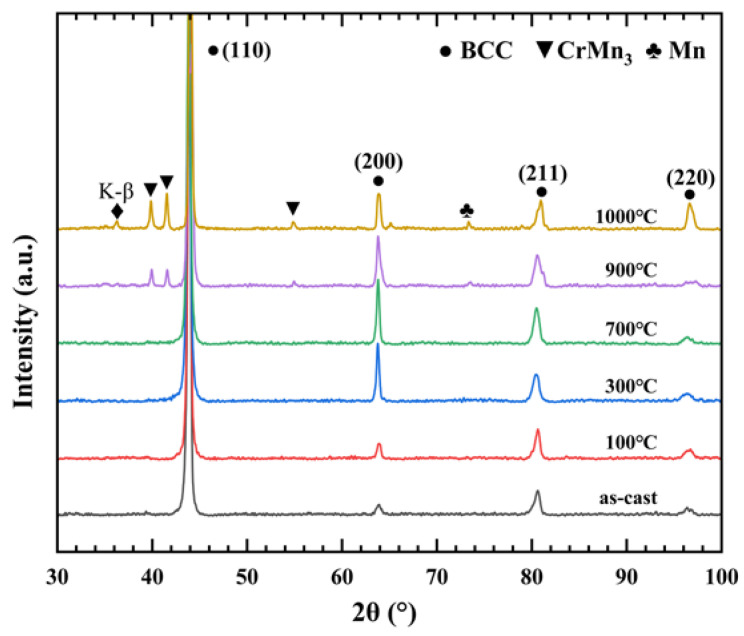
The XRD patterns of the V−Cr−Mn alloy at different annealing temperatures.

Figure 2 shows the XRD results for the V−Cr−Mn alloy as a function of annealing temperature. The diffraction peaks corresponding to the locations of BCC were confirmed by JCPDS cards #19-0797. The as-cast alloy showed a single BCC structure and maintained the BCC structure from 100 °C to 800 °C, with CrMn_3_ precipitates appearing at 900 °C and 1000 °C in V−Cr−Mn. The crystallite size of the phase corresponding to CrMn_3_ precipitates was about 29.4 nm at 900 °C and 43.7 nm at 1000 °C. The peak at about 72.5° corresponded to Mn segregation. The peak of K-β at 1000 °C in the figure was the superlattice peak caused by the instrument and had no relationship with the alloy itself. Combined with the SEM results, we observed that segregation in the alloy would eventually lead to the formation of CrMn_3_ precipitates.

Combining the SEM and XRD results, we observed that the vacancies were closely related to the migration of Cr and Mn solutes; thus, the formation of CrMn_3_ precipitates could be analyzed through the interactions between the vacancies and the solutes.

**Figure 3:**
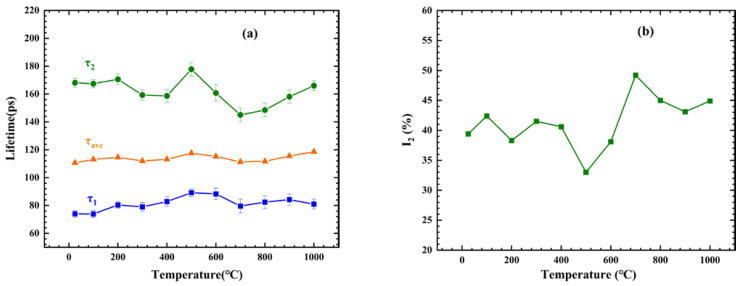
(**a**) Positron annihilation lifetime *τ*_1_, *τ*_2_, and average positron lifetime *τ_ave_* values; (**b**) the *I*_2_ intensity trapped at vacancies at different annealing temperatures.

The positron annihilation lifetime spectra were used to analyze the intermediary role of the vacancies. The samples were isochronally annealed in a temperature range up to 1000 °C, with steps of 100 °C, while the positron lifetime was measured after each annealing step. Additional analysis was performed by decomposing the positron lifetime spectra into two individual components. The experimental data consisted of the lifetime related to positron annihilation from the bulk (*τ*_1_), the lifetime of the positron trapped in the vacancy defects (*τ*_2_), the average lifetime (*τ_ave_*), the intensity of *τ*_2_ (*I*_2_), and the error. Figure 3 shows the decomposition of the lifetime spectra and the average position lifetime as a function of the annealing temperature, where parameter *τ*_1_ reflects the positron annihilation of delocalized positrons. The evolution of vacancies could be obtained from the analysis of τ_2_, and parameter *I*_2_ reflects the relative vacancy concentration that trapped positrons [20]. The average lifetime (*τ_ave_*) values calculated from the weighted averages of *τ*_1_ and *τ*_2_ are shown in Figure 3 and given by [21]:

(1) $$\tau_{ave}=\tau_{1}\times I_{1}+\tau_{2}\times I_{2}$$

Subsequently, *τ_b_* was calculated for this alloy, which reflected the defect-free bulk lifetime, and it is given by [21]:

(2) $$\tau_{b}=\frac{\tau_{1}\times \tau_{2}}{\tau_{1}I_{2}+\tau_{2}I_{1}}$$

When the average lifetime for the V−Cr−Mn alloy was significantly higher than the calculated bulk lifetime, the alloy contained vacancies as positron trapping sites. Using Formula (2), the *τ_b_* of the as-cast sample was calculated as 96 ps. Additionally, during the whole annealing process, the corresponding *τ_b_* values at each temperature in order were 98 ps, 101 ps, 100 ps, 103 ps, 108 ps, 107 ps, 103 ps, 104 ps, 106 ps, and 106 ps.

Positron annihilation contained significant and detailed information on the electronic structure to characterize the vacancy defect types and precipitates. This information could be extracted by the measurement of the coincidence Doppler broadening spectra.

**Figure 4:**
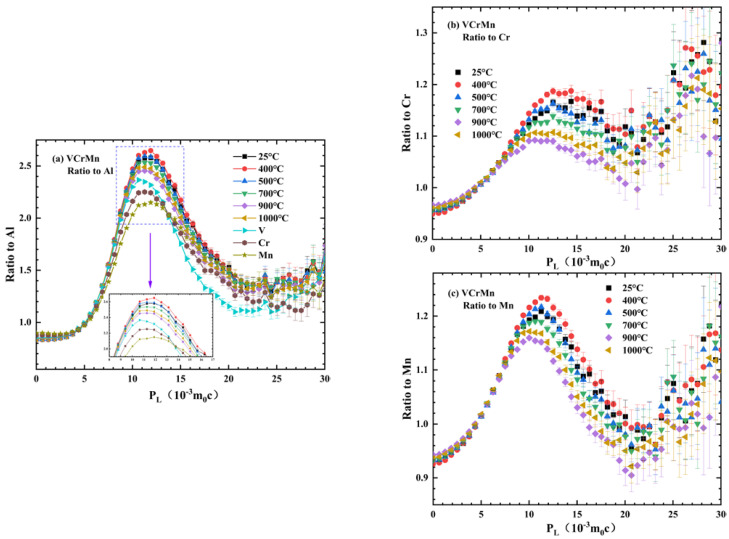
CDB ratio curves of V−Cr−Mn annealed at different temperatures to (**a**) pure Al, (**b**) pure Cr, and (**c**) pure Mn.

To identify the circumstantial characteristics of segregation and the CrMn_3_ precipitates, the momentum distribution of the core electrons was measured by CDB [22,23,24]. By taking the count of each track in the broadening spectra of the well-annealed, pure Al as a reference, each count in the Doppler broadening spectrum was compared with the obtained ratio curves for the V−Cr−Mn alloy, as shown in Figure 4a [25], and the CDB ratio curves of V−Cr−Mn relative to Cr and Mn are plotted in Figure 4b,c.

The CDB spectrum reflected the momentum distribution information. The low-momentum region ($|P_{L}|\leq 3\times 10^{-3}\;m_{0}c$) in the electron momentum distribution reflected information regarding the annihilation of positrons and valence electrons, and the high-momentum region ($|P_{L}|>3\times 10^{-3}\;m_{0}c$) exhibited the characteristic signals of the elements through positron annihilation with the core electrons [26]. Since the extracted from the CDB spectra electron momentum distribution spectra are area-normalized, the low- and high-momentum regions are correlated. When the probability of positrons trapped by vacancies was lower, the amplitude of the peak that originated from annihilation with core electrons was higher [27,28]. The ratio curves shown in Figure 4a–c in the high-momentum region magnified the information of positron annihilation with the core electrons of Al, Cr, and Mn.

If the alloy contained defects such as vacancies, namely the regions with lower than average density, it had the potential to attract positrons, and positrons could be trapped [10]. From the as-cast V−Cr−Mn alloy shown in Figure 3a, it was observed that the *τ*_1_ of V−Cr−Mn was about 74 ps, *τ*_2_ was close to 168 ps, and *τ_ave_* was about 110 ps. Our calculation using Formula (2) revealed that *τ_ave_* was higher than the *τ_b_* (96 ps) of V−Cr−Mn. Moreover, *τ*_2_ was higher than the lifetime where positrons were annihilated in the Cr monovacancies (150 ps) but a little lower than that in the Mn monovacancies (175 ps), indicating the existence of positron capture sites, such as vacancy defects [29,30].

As shown in Figure 3, there was no obvious change in *τ*_2_ and *I*_2_ from 25 °C to 200 °C, illustrating that the migration of inherent vacancies in the alloy was not significantly affected by heat treatment. This indicated that these vacancies in the as-cast samples were confined in the alloy due to the presence of Mn segregation. At 300 °C, we observed a decrease in *τ*_2_, and these confined vacancies migrated during heat treatment. Notably, these vacancies combined with solute atoms could form vacancy-solute complexes [31]. Therefore, the vacancies could regulate the migration of solute atoms. At 300 °C, a vacancy-related lifetime of 159 ps was obtained, which was much shorter than the value (170 ps) obtained at 200 °C. This lifetime value was also lower than the calculated value of monovacancy Mn (175 ps) [30], which potentially corresponded to the vacancies at the interface between Mn segregation and the matrix [32]. The increase in *I*_2_ represented an increase in the number of vacancies, which indicated that many solute Mn atoms migrated and were regulated by these vacancies. This corresponded to the idea that the Mn-segregated region dissolved into the matrix. As shown in Figure 4a, the characteristic peak of Mn was at about $11.9\times 10^{-3}\;m_{0}c$, and the characteristic peak of Cr was at about $11.3\times 10^{-3}\;m_{0}c$. The ratio curves with temperatures ranging up to 400 °C showed a characteristic peak similar to Mn at about $11.9\times 10^{-3}\;m_{0}c$, indicating that the positrons annihilated with the Mn core electrons, while the curves gradually deviated from the Mn curve with an increase in temperature, reflecting the dissolution of the Mn-segregated region.

When the relevant information of vacancies in the sample changed, the value and intensity of positron annihilation lifetime were affected. From 400 °C to 500 °C, as shown in Figure 3a,b, *τ*_2_ increased, and *I*_2_ decreased, indicating that the vacancies gathered to form clusters. The *τ*_2_ at 500 °C was 177 ps, which was close to the 175 ps lifetime where the positrons annihilated in the Mn monovacancies. When the vacancies gathered to form clusters, Mn gradually gathered, which was conducive to the formation of the Mn-segregated region. Mn segregation could provide core electrons as positron annihilation sites. Hence, the decline in the ratio curves shown in Figure 4c could be ascribed to positron annihilation with the core electrons of Mn, when Mn segregation occurred.

From 500 °C, with an increase in the annealing temperature, the sharp decrease in *τ*_2_ could be ascribed to the migration and recovery of vacancy defects, as shown in Figure 3a. However, there was an interface between the precipitates and the matrix in the V−Cr−Mn alloy, which could trap positrons; hence, the sharp increase in *I*_2_ was ascribed to the relative increase in vacancy content at the interface [31]. As a result, the positron lifetime parameters were related to the evolution of segregation and vacancy defects [33]. The signals of positron annihilation with core electrons could reflect the change in the chemical environment regarding segregation [34]. With the continuous decrease in the peak amplitude from 500 °C, the density of Cr and Mn in the matrix decreased, and segregation related to Cr and Mn gradually occurred, which was consistent with the PAL results. [35,36]. At 700 °C, the *τ*_2_ value was 146 ps, which was close to the 150 ps lifetime value where positrons annihilated in Cr monovacancies, indicating the formation of Cr clusters. As shown in Figure 1c, we observed Mn and Cr segregation, and the Cr clusters induced Cr segregation. Therefore, the process between 500 °C and 700 °C corresponded to the occurrence of Mn and Cr segregation. As shown in Figure 2, CrMn_3_ precipitates were observed at 900 °C and 1000 °C, which indicated that the basis of CrMn_3_ precipitate formation was Cr and Mn segregation. From 700 °C, *τ*_2_ increased, indicating that the size of vacancy defects at the interface increased and that the coherency of the interface decreased between the CrMn_3_ precipitates and the matrix. Therefore, this could be ascribed to the process of precipitate aggregation and growth. In general, positron trapping would occur at the CrMn_3_ precipitates, leading to Mn and Cr signals in the V−Cr−Mn alloy [37,38,39]. Therefore, the CrMn_3_ precipitates were the main positron capture sites during annealing. Additionally, the peaks of the ratio curves from 700 °C, as shown in Figure 4b,c, moved toward the low-momentum direction, illustrating that the positrons were trapped by CrMn_3_ precipitates, which was consistent with the PAL results [40].

## 4. Conclusions

The evolution of vacancies, Mn and Cr enrichments, and the formation of CrMn_3_ precipitates in a V−Cr−Mn medium-entropy alloy were investigated using PAS, SEM-EDS, and XRD analyses. We found that Mn segregation occurred in the as-cast V−Cr−Mn alloy, and the segregated regions gradually dissolved into the matrix from 400 °C, while CrMn_3_ precipitates were observed at 900 °C and 1000 °C. The PAL results showed that the vacancies gathered to form clusters, mediating the migration of Cr and Mn solute atoms to clusters. The solute clusters induced Cr and Mn segregation and finally aggregated and grew in CrMn_3_ precipitates. The crystallite size of the phase corresponding to CrMn_3_ precipitates was about 29.4 nm at 900 °C and 43.7 nm at 1000 °C. In CDB results, we observed a decrease in the amplitude of the characteristic peak in the high-momentum region and a sharp decrease in the number of vacancy defects, which could be ascribed to the migration and recovery of vacancies. The characteristic peak moving to the low-momentum direction could be attributed to the formation of CrMn_3_ precipitates and the increase in vacancy defects at the interface.

## Data Availability

Not applicable.
